# Tuning the Elastic Properties of Polymer Networks Based on a Selected Biphenyl Epoxy Precursor by Altering the Hardener—Thermal and Dielectric Approach

**DOI:** 10.3390/ma19071358

**Published:** 2026-03-29

**Authors:** Magdalena Włodarska, Lidia Okrasa, Beata Mossety-Leszczak

**Affiliations:** 1 Institute of Physics, Lodz University of Technology, Wólczańska 217/221, 93-005 Lodz, Poland; 2 Department of Molecular Physics, Lodz University of Technology, Żeromskiego 116, 90-924 Lodz, Poland; lidia.okrasa@p.lodz.pl; 3 Department of Industrial and Materials Chemistry, Rzeszow University of Technology, al. Powstańców Warszawy 12, 35-959 Rzeszow, Poland; mossety@prz.edu.pl

**Keywords:** epoxy resins, dielectric spectroscopy, differential scanning calorimetry, curing, glass transition, hardeners

## Abstract

Epoxy materials are an important class of thermosets whose properties strongly depend on the used formula, the curing parameters, and many available hardeners. Achieving desired properties such as enhanced thermal stability, extended lifetime, or self-regeneration requires selecting suitable precursors and carefully tuning curing conditions. In this work, a selected biphenyl epoxy precursor was used as a model compound to assess whether using different hardeners could be an effective factor in tailoring the elasticity of cured epoxy networks. We employed two chemically distinct hardeners—4,4′ diaminodiphenylmethane (DDM) and suberic acid—to generate materials with markedly different final properties. For instance, the glass transition temperature *T*_g_ varied within a range of over 35 °C. Two complementary experimental techniques were used in this paper to establish the optimal curing parameters: differential scanning calorimetry (DSC) and broadband dielectric spectroscopy (BDS). Both techniques supported tracking of changes in the mixture while curing and enabled determination of *T*_g_ in the obtained products. Dielectric relaxation spectroscopy revealed various molecular motions (α, β, and γ-processes) occurring in different phases, especially in glass-forming solids. BDS is therefore a good tool for testing new organic materials. The analytic route used in this work, based on a combination of calorimetric and electrical approaches, enables precise adjustment of the curing parameters to a specific hardener and helps verify the effects of using different hardeners on the elastic properties of the product. This allows the creation and modification of epoxy matrices towards modern materials, such as composites with self-healing properties or enhanced thermal stability.

## 1. Introduction

### 1.1. Designing Epoxy Resins Through Structure and Function

Development of new technologies demands smart materials that combine a series of specific physical properties [1,2,3]. Polymeric materials, including epoxy resins, offer considerable flexibility in tailoring their properties [4,5,6]. Straightforward synthesis and structural tunability (by replacing hardeners, adding elasticizing or reinforcing agents, and more) contribute to the broad commercial relevance of such resins [7,8]. Epoxy resins have excellent adhesive properties, enabling their application to various surfaces as paints, glues, or insulation layers [4,5,6]. Cured epoxy resins find applications in electrical engineering and electronics as materials with high specific resistance and low dielectric loss [9]. Epoxy matrices can also be utilized for the creation of novel composites with self-healing properties [10,11,12]. One possible repair mechanism involves thermal stimulation of the flow of the material and gluing the crack surfaces [13,14]. During the glass-to-rubber transition, polymer chains gain substantial mobility, allowing them to re-entangle after physical separation [14]. Reversible, thermoactivated elastic materials offer another route for self-healing [15]. Such applications require systems with precisely controlled glass transition temperatures and elasticity.

Obtaining resins and resin-based composites with prescribed properties depends on the precise selection of the hardeners and the adjustment of the curing conditions [16,17,18,19,20]. Many commonly used epoxy hardeners yield rigid, brittle materials with high *T*_g_. For example, curing a common DGBEA resin with aromatic amines takes place at high temperatures, yielding products with *T*_g_ as high as 200 °C [21]. A significant reduction in the glass-transition temperature for the same resin can be achieved with other types of hardeners, such as anhydrides [22]. However, acid anhydrides with different structures (e.g., maleic, succinic, phthalic, endomethylenetetrahydrophthalic, pyromellitic, or benzophenonetetracarboxylic) do not always meet expectations. They are often volatile or prone to sublimation at elevated temperatures, creating health hazards. They may also exhibit other undesirable properties, such as partial decarboxylation during curing, with end effects such as incomplete curing, or product swelling from the release of carbon dioxide [23]. Another strategy used commercially for enhancing the quality of products in chemically and thermally demanding applications, which still require a degree of flexibility, is the incorporation of plasticizers. However, the effectiveness of this method is limited, as it often diminishes both the durability and the heat resistance of the final cured materials. On the other hand, the fillers employed so far generally raise the material’s glass-transition temperature and increase its rigidity. We anticipate that choosing appropriate hardeners or modifying the resin could be a better way towards a material with a lower *T*_g_ and improved elasticity of the cured resin. Potential applications of the obtained resin can be sought in using it to create advanced polymer composites with reduced influence of the active filler on other material properties, such as its rigidity or *T*_g_.

In designing the curing process for a selected resin, a suitable hardener can be chosen from the many available options to achieve the desired properties of the final product. This choice might focus, e.g., on mechanical properties or thermal resistance (essential for durability and quality features of the material). It may also target the glass transition temperature at which the polymer shifts from a brittle material to a softened, elastic one [20]. The hardener, together with the curing temperature and duration, is the key parameter that must be optimized to obtain the desired material properties. And so, as also demonstrated in this work, aromatic amines as hardeners produce durable, thermally and chemically stable, rigid, and high-*T*_g_ materials, although they may be prone to mechanical brittleness. In contrast, acidic hardeners yield more elastic products whose glass transition temperature is strongly dependent on the curing conditions. Consequently, systems cured with such hardeners require precise determination of the temperature range in which the final material can be used without changes in its properties. For this reason, dicarboxylic aliphatic acids (such as suberic acid) are less commonly used as hardeners. It is somewhat unfortunate, as selecting acids with different aliphatic chain lengths and appropriately adjusting the curing conditions makes it possible to obtain materials with a finely controlled *T*_g_ range. This sets certain constraints on possible applications of epoxies cured with acids, but can also be an advantage in building advanced, thermally activated materials. A good example here can be organogels created from such matrices and used for thermally reversible light scattering films [15].

In our earlier research, we investigated the influence of the molecular structure of epoxy precursors and of the curing conditions (adjusted to match the phase transitions occurring in the precursors) on the structure and physical properties of the cured resins. We studied precursors with different rigid cores (di-, tri-, and tetra-aromatic) as well as varying lengths of flexible aliphatic chains, using several types of crosslinking agents. Part of this research was summarized in a review article [24]. Most recently, we sought increasing network flexibility (and thus lower *T*_g_) by introducing precursors with long aliphatic chains and non-terminal epoxy groups. However, there is an indication that a comparable effect can also be achieved by reducing the rigidity of the curing agent [25,26].

In the present work, we investigate—for the first time—an epoxy precursor with a biphenyl core (and C8 terminal alkyl chains), expecting a product with increased elasticity. The selected epoxy resin was complemented with two types of hardeners—a diaromatic amine and a dicarboxylic acid. Our aim was to create both stiff networks and flexible ones (with *T*_g_ close to the target working temperatures), to demonstrate the broadest possible potential of DKUU resin.

### 1.2. Combining DSC and BDS for Characterization of Polymer Networks

The influence of the chosen hardeners on selected properties of the cured products is established through examination with differential scanning calorimetry (DSC) and comparing the results with information obtained from broadband dielectric spectroscopy (BDS). The combination of both analytic techniques facilitates precise adjustment of the curing conditions to each specific hardener. Initial calorimetric studies (DSC) carried out upon heating the reagents enable the determination of the temperature range in which the curing process takes place [27,28,29]. But establishing time bounds of the whole curing reaction already requires additional experiments. In practice, the substrates are cured in isothermal conditions, and the speed of curing depends on the chosen temperature. Moreover, temperature selection affects the physical properties and the structure of the final product. Electrical methods proved to be useful for in situ observations of the reaction progress, because both the electric conductivity and the electric permittivity are highly sensitive to structural or phase changes. In the domain of organic materials, monomers and polymers included, the calorimetric measurements (usually conducted in heating-cooling cycles) enable observation of phase transitions. Such transitions—specifically, the crystallization and vitrification processes—depend strongly on the rate of temperature change and the thermal history of the material. In the phase transition regions, DSC curves exhibit characteristic endo- or exothermic peaks, whereas the vitrification region typically shows only some deviation from the baseline caused by large changes in thermal capacitance, which is not always easy to delineate. For that reason, applying techniques such as dielectric spectroscopy (BDS) is an effective complementary approach to obtain a more complete picture. This technique is highly sensitive to any changes in the material’s structure, including phase transition or glass transition, and it relies on measurements of the complex dielectric permittivity in a wide range of frequency and temperature [30,31,32,33]. The real part of the dielectric permittivity, associated with changes in the arrangement of dipole moments in the dielectric material, changes significantly in the vicinity of phase transitions. The imaginary part of the dielectric permittivity allows monitoring of molecular motions, including the motion of chains and rigid parts of the molecules, and it allows capturing changes in the molecular mobility that occur during phase transitions and the glass transition. These motions are visible as relaxation processes, and they depend on the presence of dipolar groups in the molecules, as well as the whole microscopic structure of the material, which can be correlated with macroscopic physical properties. Appearance or disappearance of a specific relaxation mode may reflect a phase transition or a glass transition. In most cases, the observed relaxation processes are readily identifiable and easy to follow. In polymeric materials, a series of relaxations is usually observed. These processes are denoted with Greek letters α, β, and γ. The α-process has been attributed to cooperative relaxation mode related to the motion of polymer chain segments and structural changes, while the β and γ-processes are generally interpreted in terms of local rotational motions of molecular fragments or polar groups. This interpretation appears in many experimental studies on various classes of polymers [30,31,32,33]. Vanishing of the α-mode during the cooling route enables identification of the rubber-to-glass transition in the analyzed sample, and makes some predictions about fragility, which is also correlated with certain changes in the mechanical properties of the material. In practice, from the α-mode, one can obtain the glass transition temperature. To analyze all the relaxation processes, a semi-empirical Havriliak–Negami (HN) formula with DC conductivity is often used [30,34]:

(1) $$\varepsilon^{*}(\omega )-\varepsilon_{\infty }=-i\frac{\sigma_{0}}{\varepsilon_{0}\omega^{s}}+\sum_{k}\frac{∆\varepsilon_{k}}{(1+(i\omega \tau_{k}{)}^{\alpha_{k}}{)}^{\beta_{k}}}$$

Using this formula, we can describe each individual relaxation process with a series of parameters and track how they evolve with changes in the temperature. The relaxation times *τ_k_*, the shape parameters *α_k_*, *β_k_*, and the DC conductivity *σ*_0_ together form a useful framework for a broad analysis of the observed processes. The remaining components of the H-N formula are the electric permittivity of vacuum (*ε*_0_), the high-frequency bound of *ε*′ (*ε*_∞_), the radial frequency (*ω* = 2π*f*), and the dielectric strength (Δ*ε*—the difference between the real parts of the high-frequency and low-frequency bounds of the electric permittivity, associated with the given relaxation process); the *s* exponent (*s* ≤ 1) accounts for any non-ohmic effects.

In polymers, the α-relaxation (linked to the glass transition) is typically the slowest dynamic process, so it appears at the lowest measurement frequencies or, equivalently, at the highest measurement temperatures [35,36]. The dependence of the relaxation time on the temperature for the α-process can be described with an empirical function of the temperature *T* proposed by Vogel, Fulcher, and Tamman (VFT) [31,32]:

(2) $$\ln(\frac{1}{\tau })=\frac{-DT_{V}}{T-T_{V}}+\ln(\frac{1}{\tau_{0}})$$

while the relaxation times of the remaining processes usually obey the Arrhenius relation:

(3) $$\ln(\frac{1}{\tau })=\frac{-E_{a}}{kT}+\ln(\frac{1}{\tau_{0}})$$

with the characteristic activation energy *E_a_*. The *D* parameter in the VFT formula describes a deviation of the temperature dependence of the relaxation time *τ* from the Arrhenius law. *T_V_* is the so-called Vogel temperature, which is sometimes is identified as the ‘ideal’ glass transition temperature [37]. The *D* parameter can also be directly related to a so-called ‘fragility index’ *m*, through the following relation:

(4) $$m=\frac{{DT}_{0}}{2.3\cdot T_{g}{\left(1-T_{0}/T_{g}\right)}^{2}}$$

with the fragility index defined as [38]:

(5) $$m={\left[\frac{d\left({log}_{10}\tau \right)}{d\left(T_{g}/T\right)}\right]}_{T=T_{g}}$$

Another parameter that can be calculated is the apparent activation energy *E_a_*′ at *T*_g_ using the following formula [39]:

(6) $$E_{a}\prime (T_{g})=\frac{\;RDT_{V}}{(1-\;\frac{T_{V}}{T_{g}}{)}^{2}}$$

where *R* is the universal gas constant and equals 8.314 J mol^−1^ K^−1^.

Dielectric techniques enable in-situ monitoring of curing reactions, including those in epoxy systems. The progress of curing can be followed by observing changes in the shape or intensity of dielectric relaxation peaks—such as their broadening or reduced amplitude—or by tracking shifts in their position within the frequency domain [40,41,42]. Low DC conductivity favors the use of complex permittivity for analysis of dielectric data, whereas measurements utilizing the direct current are more effective in high-conductivity regimes. In our case, direct electric measurements can be applied to in situ monitoring of the curing reaction in epoxy resins [25]. The progress of curing can be tracked by directly recording changes in the DC conductivity.

## 2. Materials and Methods

### 2.1. Synthesis of Propane-2,2-diylo-4,4′-diphenyl Bis(10,11-epoxyundecanoate)—DKUU

Commercial reagents used in the synthetic route were supplied from Sigma-Aldrich/Merck (Steinheim, Germany): 2,2-bis(p-hydroxyphenyl)propane—bisphenol A (99%), tetrabutylammonium bromide (≥99%), 10-undecenoic acid; Fluka Chemie GmbH (Buchs, Switzerland): N,N′-dicyclohexylcarbodiimide, 4-(N,N-dimethylamino)pyridine, *m*-chloroperbenzoic acid (70%); Lancaster Synthesis GmbH (Kehl, Germany): perchloric acid (70%); Avantor Performance Materials Poland S.A. (Gliwice, Poland): methanol, sodium chloride, chloroform, dichloromethane, anhydrous magnesium sulfate, sodium sulfite, sodium bicarbonate. All the reagents purchased were of analytic grade unless otherwise noted and were used without further purification.

The first stage of the synthesis was the esterification reaction of bisphenol A (4,4′-(propane-2,2-diyl)diphenol) with 10-undecenoic acid according to the Steglich method [43]. N,N′-dicyclohexylcarbodiimide (DCC) was used as a dehydrating agent to shift the equilibrium of esterification, and 4-(dimethylamino)pyridine (DMAP) was the reaction catalyst. DCC is well soluble in the reaction mixture and after reaction with water forms a urea derivative N,N-dicyclohexylurea (DHU), which precipitates from the solution in the form of an easily removable crystalline product.


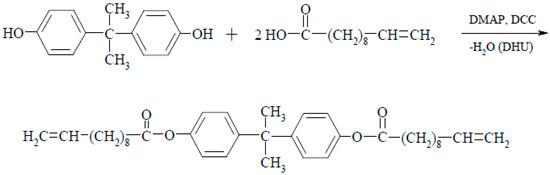


Appropriate amounts of bisphenol A (2.28 g, 0.01 mol), 10-undecenoic acid (3.68 g, 0.02 mol), and dichloromethane as a solvent were introduced into a 250 cm^3^ round-bottom flask equipped with a magnetic stirrer, thermometer, and air condenser. Then, weighed DMAP (0.0977 g, 0.0008 mol) and DCC (4.54 g, 0.022 mol) were added to the reaction mixture. The esterification was carried out for 24 h at room temperature, after which the precipitated DHU was separated, and the solvent was distilled using a rotary evaporator. The crude product was purified by crystallization from 40 cm3 of methanol. 4.6 g of DKU product was obtained (a yield of 82%).

**^1^H-NMR** (CDCl_3_, δ(ppm), *J*): 7.21 (4H, d, *J* = 8.7 Hz, aromatic), 6.96 (4H, d, *J* = 8.7 Hz, aromatic), 5.80 (2H, m, –CH=CH_2_), 4.96 (4H, m, –CH=CH_2_), 2.53 (4H, m, CH_2_-COO), 2.04 (4H, m, –CH_2_–CH=CH_2_), 1.73 (4H, m, CH_2_–CH_2_–COO), 1.42 (20H, m, (CH_2_)_5_), 1.36 (6H, t, C–(CH_3_)_2_).
**FT-IR** (KBr, ν(cm^−1^)): 3080, 1603–1465, 1017, 848 (aromatic), 2964–2851 (CH_2_), 1750 (C=O), 1279–1081 (C–O), 1641 (CH=CH_2_).

Then, the oxidation of unsaturated bonds in the resulting product DKU was carried out. An effective and selective method known in the literature was chosen, in which *m*-chloroperbenzoic acid is used for epoxidation.


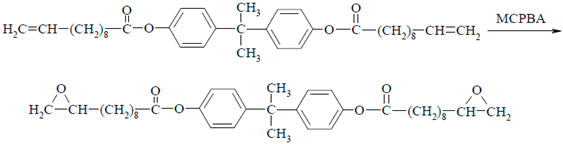


DKU (3.5 g, 6.25·10^−3^ mol), *m*-chloroperbenzoic acid (70%) (3.7 g, 1.5·10^−2^ mol), and 60 cm^3^ dichloromethane were introduced into a 250 cm^3^ round-bottom flask. Oxidation with MCPBA was carried out at room temperature for seven days. After completion of the reaction, the resulting precipitate of *m*-chlorobenzoic acid was filtered off, and the filtrate was washed successively with a 5% aqueous solution of Na_2_SO_3_, 5% aqueous solution of NaHCO_3_, and a saturated NaCl solution. The organic layer was dried with anhydrous MgSO_4_. After removal of the drying agent and solvent, the crude product was recrystallized from 100 cm^3^ of methanol. 2.8 g of DKUU (76% yield) was obtained.

**^1^H-NMR** (CDCl_3_, δ(ppm), *J*): 7.20 (4H, d, *J* = 8.7 Hz, aromatic), 6.96 (4H, d, *J* = 8.8 Hz, aromatic), 2.90 (2H, m, CH of epoxy), 2.74 (2H, m, CH_2_ of epoxy), 2.60 (4H, m, CH_2_–COO), 2.48 (2H, m, CH_2_ of epoxy), 1.75 (4H, m, –CH_2_–CH_2_–COO), 1.55 (4H, m, –CH_2_-epoxy group), 1.41 (20H, m, (CH_2_)_5_), 1.38 (6H, t, C–(CH_3_)_2_).
**FT-IR** (KBr), ν (cm^−1^): 3061, 1601–1465, 1017, 851 (aromatic), 2960–2850 (CH_2_), 1742, (C=O), 1288–1082 (C–O), 925 (epoxide).

### 2.2. Sample Preparation for Curing

**Figure 1:**
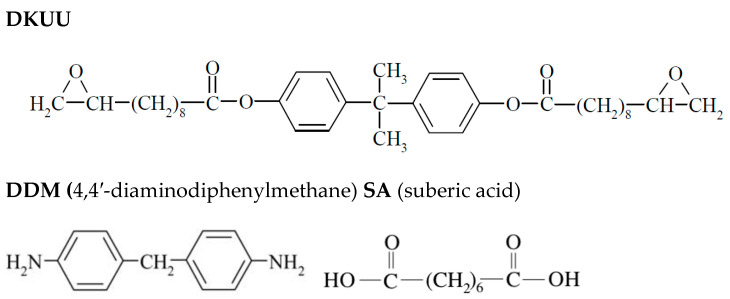
The molecular structure of the investigated compound (DKUU) and the hardeners (amine and acid). Melting temperatures: DDM—91 °C; SA—142 °C.

The synthesized precursor (DKUU) was separately mixed with each of the chosen hardeners—DDM or SA—in stoichiometric ratios equivalent to a 1:1 molar ratio of epoxy groups to the amine or acid groups, respectively. Both hardeners were purchased from Sigma-Aldrich/Merck and were used as received, without further purification. All the mixtures were prepared and pulverized shortly before commencing the thermal and electric measurements. Reports on the curing reaction kinetics in similar systems can be found in references [44] for DDM and [45] for SA. The molecular structures of the monomer and both hardeners are shown in Figure 1.

The curing conditions for all the investigated products were determined during the investigations, which are reported and discussed in detail in the next section of the paper. In particular, it was determined that 150 °C for 3 h is a sufficient and appropriate curing temperature for both systems, although the acid-cured system (DKUU/SA) additionally requires post-curing at 250 °C.

### 2.3. Experimental Methods

Dielectric permittivity of neat monomer and the cured compositions was measured across a frequency range of 10^6^ to 10^−1^ Hz. A Novocontrol Alpha high-resolution dielectric analyzer combined with a Quatro cryosystem (Novocontrol Technologies GmbH, Montabaur, Germany) was used under isothermal conditions with temperature stability of 0.1 K. The measurements were performed using sandwich-type cells made of parallel metal electrodes (*d* = 10 mm) and silica spacers (50 µm), with analyzed samples placed inside the cells.

Isothermal curing was monitored electrically using a setup that included a Manson SDP 2803 programmable digital switching-mode DC regulated power supply (Manson Engineering Industrial Ltd., Hongkong, China) connected to a Fluke 8808A digital multimeter (Fluke Corp., Everett, MA, USA). The system was operated through dedicated proprietary software. In this configuration, temperature accuracy of ±0.5 K was provided by a UNIPAN 660 device (Unipan, Warsaw, Poland).

The structure of the products was determined by ^1^H NMR and FT-IR spectroscopy with a Bruker Avance II Plus spectrometer (Bruker Corp., Billerica, MA, USA) operating at 500.13 MHz under a static magnetic field of 11.7 T and a Thermo Scientific Nicolet 8700 FT-IR spectrometer (Thermo Fisher Scientific Corp., Waltham, MA, USA), respectively.

The DSC setup used for thermal analysis consisted of a Mettler Toledo DSC-1 instrument (Mettler Toledo GmbH, Greifensee, Switzerland) controlled by dedicated software (STAR^e^ System), with In and Zn calibration standards also supplied by Mettler Toledo. Thermograms recording was performed at a fixed heating and cooling rate (10 K/min) in an inert atmosphere (N_2_ flow rate: 60 cm^3^/min).

## 3. Results and Discussion

### 3.1. Characterization of the Epoxy Precursor—DKUU

#### 3.1.1. Thermal Analysis

**Figure 2:**
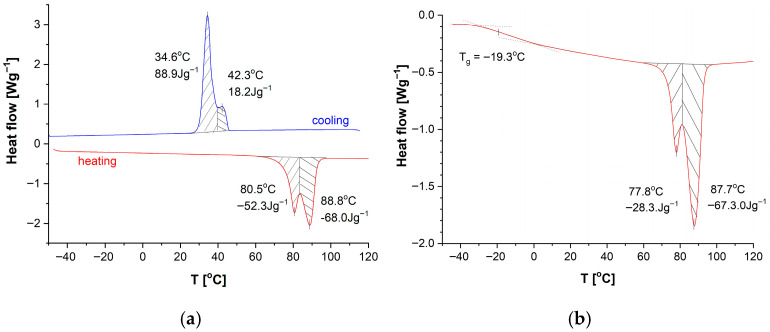
DSC thermograms of non-cured DKUU: (**a**) cooling and heating at a rate of 10 deg/min after previously melting the sample at 120 °C; (**b**) heating at a rate of 10 deg/min after previously melting the sample at 120 °C and rapidly cooling at a rate of about 25 deg/min.

The results of the standard calorimetric analysis of the DKUU monomer, conducted at the rate of 10 deg/min, are presented in Figure 2a. A crystallization process occurs at 42 °C in the cooling route, whereas its counterpart in the heating route—crystal melting—takes place above 89 °C. This temperature helps identify the compound’s melting point in mixtures, which is essential for analyzing the curing process. It is noteworthy that the crystallization process is characterized by a broad heat-flow peak with two visible maxima. Epoxy materials, including monomers, often consist of a mix of crystalline and amorphous phases. The potential presence of amorphous regions in the material is also an important aspect of the analysis. For that reason, we conducted an additional experiment with a high heat flow rate to determine the glass transition temperature of the amorphous phase. This was assessed by rapid cooling of the sample at the rate of 25 deg/min. A subsequent, detailed DSC study revealed the existence of amorphous phase content with *T*_g_ in the low-temperature region, around −19.3 °C (Figure 2b). However, even such rapid cooling revealed only a minor amorphous fraction in the material; the crystallization peak around 80 °C remained, although with a noticeable decrease of the total enthalpy (from ~52 J/g to ~28 J/g in the first sub-peak, with the other sub-peak remaining unchanged at 67–68 J/g—Figure 2). The effect is subtle in this case, and the confirmation of the glass transition temperature of the amorphous phase can only be found in the dielectric studies. These studies are of particular significance because the appearance of an amorphous phase may have a large impact on the properties of both the plain monomer and the cured products. Finally, the observed phase transitions—crystallization, melting, and vitrification—may be compared with the dielectric measurements where the phase transitions are reflected in a sudden change of the electric permittivity, and the amorphous phase is associated with a relaxation process that vanishes near the glass transition temperature.

#### 3.1.2. Dielectric Response

**Figure 3:**
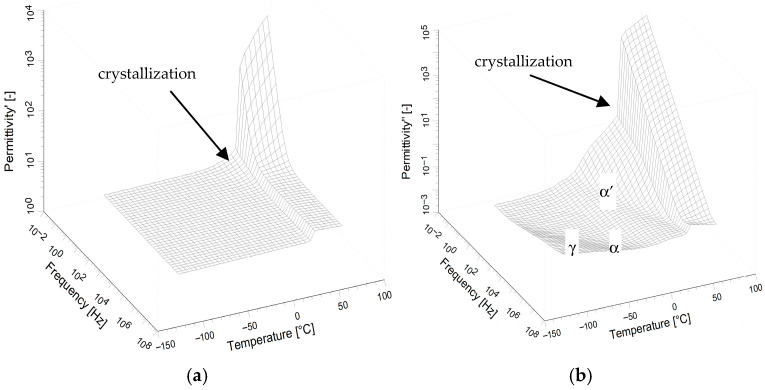
The real (**a**) and imaginary (**b**) components of the complex dielectric permittivity vs. frequency and temperature in 3D representation for neat DKUU. Cooling from 120 °C (from liquid state).

**Figure 4:**
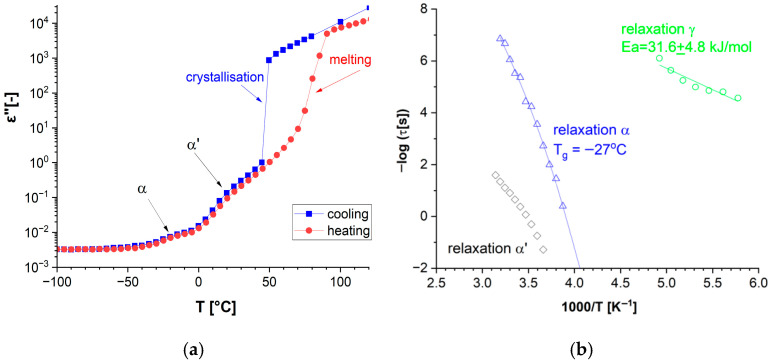
The imaginary component of the complex dielectric permittivity of DKUU in a wide temperature range, at the frequency of 1.15 Hz: (**a**) cooling from 120 °C (blue squares) and heating from −100 °C (red circles), and (**b**) the temperature dependency of the relaxation time for all processes observed in non-cured DKUU during cooling.

The dielectric behavior of neat DKUU is presented in terms of dielectric permittivity over a broad frequency/temperature range (Figure 3). The imaginary component spans several orders of magnitude over the investigated temperatures; it is therefore shown using a logarithmic scale. The liquid-solid transition is the only clearly identifiable feature in the real part of the electric permittivity (Figure 3a). Direct-current (DC) conductivity dominates the dielectric spectra at low frequencies and elevated temperatures, preventing detection of relaxation processes in the isotropic phases within the available measurement range. The plot of the imaginary part of the electric permittivity (Figure 3b) reveals a corresponding, pronounced decrease in the electric conductivity at the liquid-solid transition. This broad phase transition is consistent with the behavior observed in DSC studies (see Figure 2a). It is also clearly visible in the plot of the imaginary component versus temperature (Figure 4a), particularly the difference between the heating and cooling cycles in the phase transition temperature.

However, at temperatures far from the phase transition, the electrical parameters are almost identical for both cycles in the tested material. At temperatures below the phase transition, relaxation processes become apparent (Figure 3b and Figure 4b). The first one (α′-relaxation) is seen near the phase transition temperature in the solid phase. The occurrence of this process is correlated with a broad phase transition visible in both BDS and DSC measurements. The other process seen at a lower temperature is the α-process—most likely related to a glass transition. This distinct, although low-intensity, α-process indicates a small, but still present, glassy phase in the structure and is directly related to the transition seen in DSC measurements (see also Figure 2b). The glass transition temperature, along with the VFT fitting curve, is shown in Figure 4b, and it is in very good agreement with that determined by calorimetric methods. Another process appearing in the very low temperature range with the typical Arrhenius curve (Figure 4b) is usually related to local molecular motions and is denoted in our work as the γ-process. A similar relaxation of polar groups (most likely related to the rigid part of the bisphenol-A molecule) should also be visible in cured materials with similar activation energy, regardless of the hardener used.

### 3.2. Observation of the Curing Process

**Figure 5:**
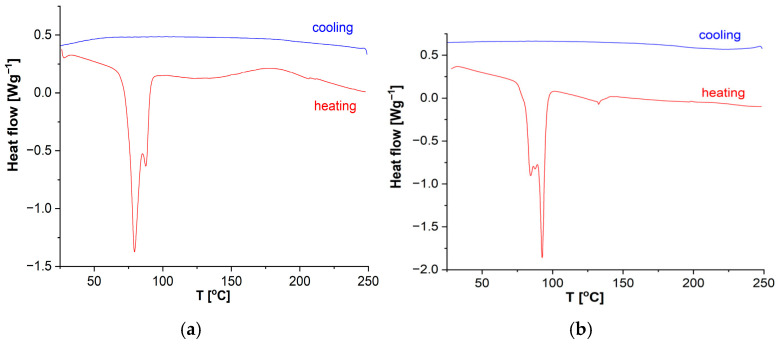
DSC thermograms of the curing process of DKUU (heating route) directly followed by a cooling route. DKUU cured with DDM (**a**) and with SA (**b**).

For DKUU, the curing requires relatively high temperatures—at least 130 °C—both with DDM and with the suberic acid (Figure 5). In the first mixture, two melting processes are initially visible. They correspond to the melting of the epoxy material and the amine. Further heating reveals a clear exothermic peak associated with the curing reaction (Figure 5a). After heating to 250 °C, no further changes occur during the cooling cycle. A similar thermogram can be observed for the second mixture (Figure 5b), but the exothermic process is now visibly weaker, and it does not exhibit a distinct peak such as that observed in the first case. Despite these differences, heating to 250 °C also leads to a full conversion of the mixture.

**Figure 6:**
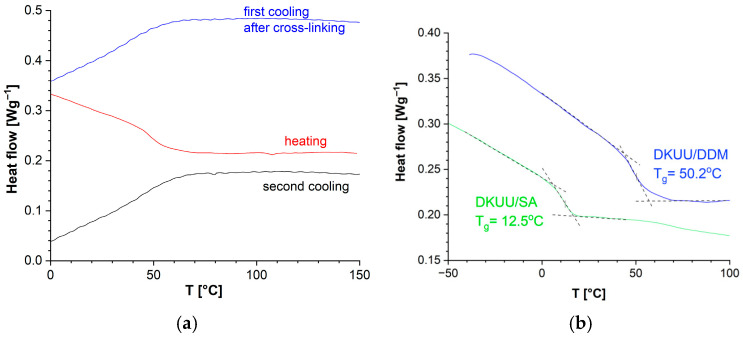
DSC thermograms: (**a**) DKUU cured with DDM, illustrating repeatability of the results; (**b**) DKUU cured with DDM and SA, showing determination of *T*_g_ from the heating data.

The DSC thermogram of subsequent heating and cooling cycles after curing is shown in Figure 6. The only process observed in the products during those cycles is the glass transition, confirming that the monomer was already fully cured during the first heating to 250 °C (see Figure 6a). Analogous curves were recorded for the acid-cured system (these graphs were included in Supplementary Materials Figure S1). A general summary of these observations is that both systems heated up to 250 °C undergo full conversion, and phase transitions observed earlier no longer appear in DSC thermograms recorded during subsequent heating and cooling cycles. The glass transition temperatures determined from repeated cooling cycles do not show meaningful variability, proving that the obtained products are thermally stable, fully cured epoxy resins. A detailed comparison of the glass transition temperatures of both mixtures is shown in Figure 6b, where a significant difference between the two products is evident in this respect.

Calorimetric observations (DSC) are conducted with temperature changes, whereas the curing process of epoxy materials is typically performed under isothermal conditions. The process of establishing the conditions for such isothermal curing usually requires balancing several competing factors. Curing at a higher temperature is faster, which may negatively affect the structure of the resulting network. For that reason, it is preferable to choose a temperature near the lower end of the applicable temperature range. On the other hand, lower temperatures require longer curing times, sometimes followed by post-curing at a higher temperature. Repeatability of the curing process and stability of the products are other concerns. As evidenced by the DSC curves (Figure 5), the curing reaction occurs at elevated temperatures—the onset of curing can be observed in the vicinity of 150 °C in both systems. However, as seen in Figure 5, the dynamics of that process are different for both hardeners (for example, there is a heat flow increase in the amine system above 150 °C, with a well-shaped maximum near 180 °C). The melting temperatures of both hardeners are also different: the amine melts in the region of 90 °C (which overlaps with the temperature range of the monomer melting in the studied system, making it difficult to clearly separate the amine and monomer peaks in the DSC plots), while the melting of the chosen acid only occurs around 140 °C. Detailed plots for the first heating cycle of the studied mixtures (with integration of the observed peaks) were included in Figure S2a,b in the Supplementary Materials. In DKUU/DDM, the exothermic curing reaction is evident, with the total enthalpy of ~116 J/g, even though the whole reaction is spread across a broad temperature range (Figure S2a). In DKUU/SA, the observed exothermic effect is much smaller, pointing to different reaction dynamics (Figure S2b). Nevertheless, the curing reaction undoubtedly takes place in this case as well, as evidenced by the lack of exo- and endothermic peaks in subsequent cooling cycles (Figure 5). The reaction dynamics in similar systems are typical for both the hardeners and were reported earlier in papers [44] for the amine and [45] for the acid hardener. The conduct of the reaction observed in the systems studied in this work is therefore consistent with the literature. Based on these observations and all the selection criteria mentioned above, we decided to choose the temperature of 150 °C as the basis for further investigations: it is still relatively low, but above the onset of the curing reaction in both systems. In the next step, we performed a few experiments to verify repeatability of curing for different samples—example plots demonstrating this are shown in Supplementary Materials (Figure S3).

**Figure 7:**
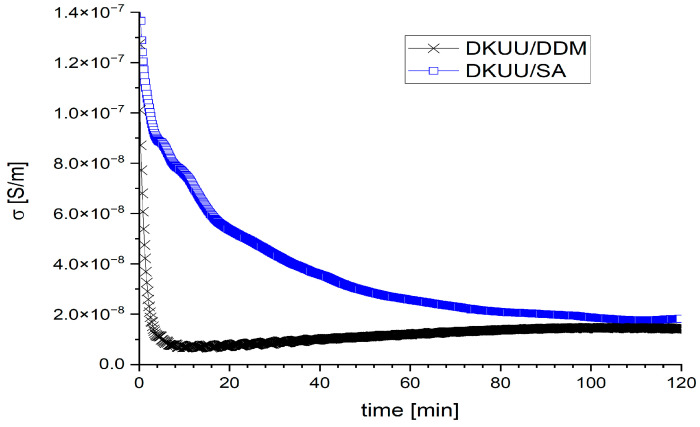
In situ observation of isothermal curing through changes in the electric conductivity with time: DKUU cured at the temperature of 150 °C with DDM and with SA.

DC electric conductivity offers an additional means of monitoring the advance of isothermal curing. Performing in-situ measurements enables continuous tracking of the conversion degree by following conductivity changes as the material cures at a fixed temperature. Such measurements additionally allow an estimation of the total curing time at a given temperature. When curing is carried out at elevated temperatures, the DC conductivity is also relatively high. A representative example of such observations is shown in Figure 7, allowing for a comparison of the conductivity of both mixtures cured at the same temperature of 150 °C. The changes occurring in the conductivity with time prove that the reaction progresses continuously over a period of at least two hours for both mixtures (at that temperature), although evolving somewhat differently. The mixture is initially in a liquid phase characterized by high conductivity, which decreases with changes in the mixture viscosity. In the case of DKUU/DDM, the conductivity drops quickly at first (which may be associated with solidification of the material) and then the reaction progresses slowly—the conductivity gradually stabilizes. In DKUU/SA, a gradual decrease in the conductivity is observed throughout the duration of the measurements. Although observations of the conductivity may help determine the point in time when the reaction almost stops at a given temperature, that temperature might not be sufficient to ascertain complete conversion of the mixture. Full information on the reaction progress that enables choosing the optimal conditions for a full cure can only be obtained by combining these observations with a calorimetric study. Therefore, additional DSC analysis was carried out for samples cured at 150 °C for 3 h. The values of *T*_g_ observed in these experiments for the amine system were identical to those shown in Figure 6a. In the studied mixture based on DKUU/SA, post-curing at 250 °C was found essential to achieve a full conversion, with heat flow peaks associated with melting of the substrates no longer appearing in DSC thermograms (see Figure 5). A fully cured product can be obtained by selecting slightly different curing conditions (like the time and temperature of curing, or the temperature and conditions of post-curing). Therefore, the cured materials also need to be examined to determine the properties of the obtained product. The curing conditions ultimately chosen for subsequent analyses of the studied mixtures and their corresponding products are compiled in Table 1. The products obtained by curing with the two hardeners are compared in the next section of the paper, which concludes with tabularized values of the glass transition temperatures (*T*_g_) as determined in both DSC and BDS approaches (Table 2).

### 3.3. Dielectric Properties of the Cured Products

**Figure 8:**
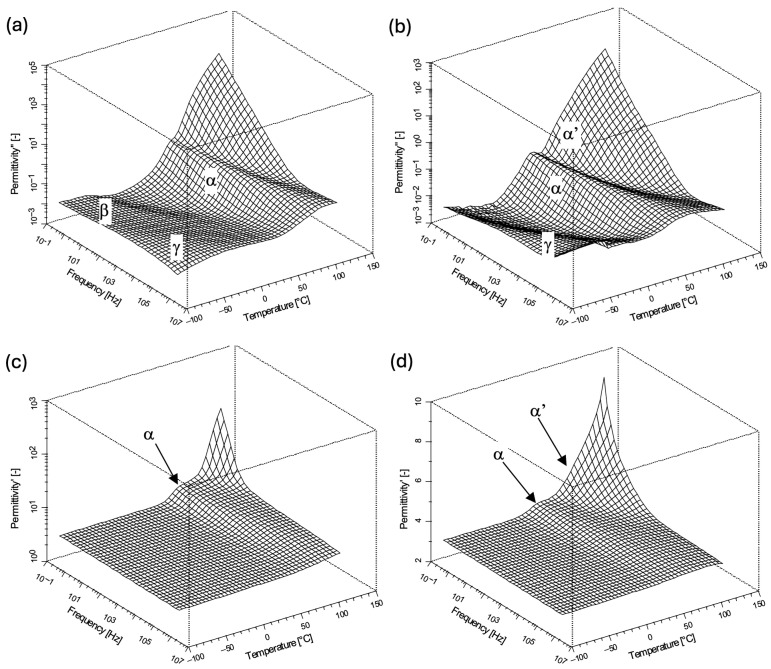
The imaginary (**a**,**b**) and real (**c**,**d**) parts of the complex dielectric permittivity vs. frequency and temperature in 3D representation for products of curing DKUU with DDM (**a**,**c**) and SA (**b**,**d**).

The final step involved comparing the dielectric responses of the two products obtained with the selected curing parameters (compiled in Table 1). Figure 8 shows the real and imaginary parts of the permittivity for both materials across a broad frequency and temperature range. The increase in the permittivity value at high temperatures, visible in all figures, is related to the increase in conductivity. In both cases, the substantial decrease in the DC conductivity—correlated with the glass transition—is clearly visible (Figure 8). In both products, as well, the α and γ-processes appear; although the electric conductivity partially overshadows the α-process in the first network, it is still observable at selected temperatures. Observation of the α-process is particularly important because it allows determining the glass transition temperature. In spite of these commonalities, clear distinctions between the two compositions are also evident when their dielectric spectra are compared.

**Figure 9:**
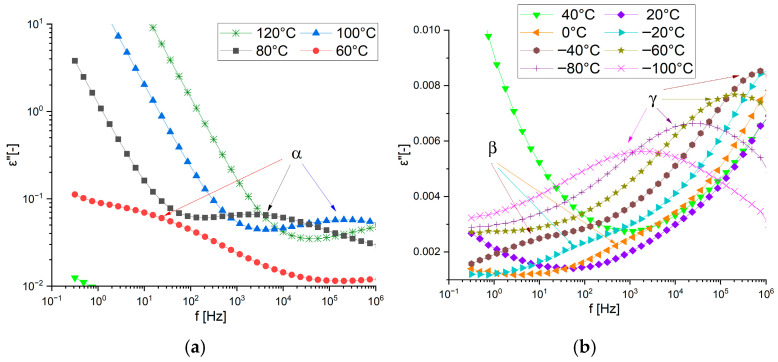
The imaginary component of the complex dielectric permittivity vs. frequency at selected temperatures for DKUU cured with DDM: (**a**) the temperature range of the α relaxation; (**b**) the temperature range of the β and γ relaxations.

**Figure 10:**
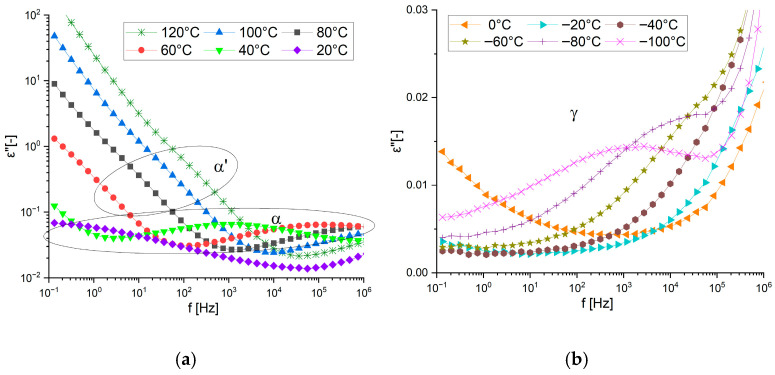
Imaginary part of the complex dielectric permittivity vs. frequency at chosen temperatures for DKUU cured with SA: (**a**) shows the temperature region of the α′ and α relaxations, (**b**) shows the temperature region of the γ relaxation.

Details of the observed relaxations are shown in Figure 9 for the amine-cured epoxy and in Figure 10 for the acid-cured product. In the first case, only the α-process is visible at high temperatures (Figure 9a), with two relaxation processes—β and γ—observed at low temperatures (Figure 9b). A low-amplitude β-process is only visible at temperatures around −10 °C in the amine-cured material. At low temperatures, the γ-process is clearly visible in both the obtained products and in the monomer. This process can therefore be associated with the rigid core of the monomer.

The appearance of the β-process and the high glass transition temperature clearly distinguish the product obtained by curing with a stiffer hardener. This represents a significant difference in the molecular dynamics of the material, which is also related to the macroscopic and mechanical properties of this product. Figure 10 shows details of the relaxation processes occurring in the acid-cured material. At high temperatures (Figure 10a), in addition to the α-process associated with the glass transition temperature, a weak α′-process is visible. This process is like the one seen in the monomer itself, but there is no crystallization process that clearly distinguishes the resulting product from the pure monomer. At low temperatures, only the γ-process is visible (Figure 10b).

**Figure 11:**
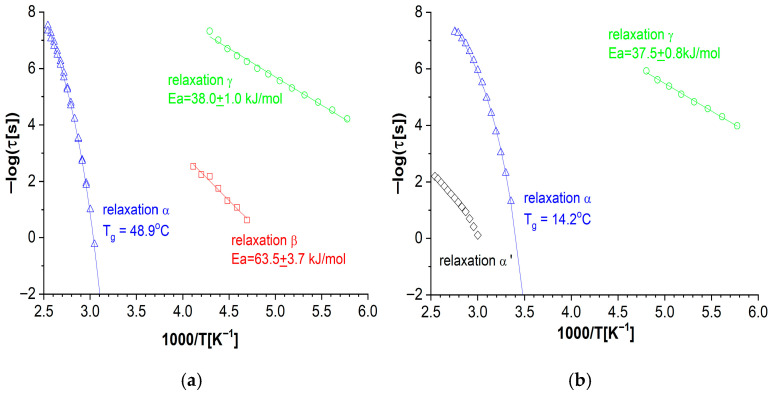
The relaxation time vs. temperature for all detected relaxations—a characteristic VFT curve for the α-process, an Arrhenius dependency for the β and γ processes: (**a**) DKUU/DDM; (**b**) DKUU/SA.

All observed processes were fitted with the H-N equation (Equation (1)) (representative fits can be found in Supplementary Materials, Figure S4). The temperature dependencies of the relaxation times obtained in this way for all the detected processes are plotted in Figure 11. These dependencies follow the Arrhenius relation (Equation (3)) for the β and γ-processes, and the VFT relation (Equation (2)) for the α-process. Within the VFT framework, *T*_g_ can be estimated by identifying the temperature at which the α-process becomes too slow to be experimentally observed; by convention, when τ^−1^ crosses the 10^−2^ Hz mark, which is equivalent to τ exceeding 100 s. Detailed VFT fitting parameters are included in Supplementary Materials (Figure S5). *T*_g_ values obtained in the dielectric study are in reasonable agreement with those from thermal analysis, as summarized in Table 2.

## 4. Conclusions

This study demonstrated that the choice of hardener type fundamentally alters the dynamics of the curing process, requiring different parameters such as the curing temperature and time, or the need for post-curing, and leading to different product properties. We used both dynamic temperature-controlled calorimetric measurements (DSC) and constant temperature electrical measurements (BDS) to obtain the precise curing parameters. The selection of the hardener is instrumental for creating materials with desirable properties, as shown in the obtained results, where the application of two different hardeners led to products with significantly different mechanical (elastic) properties.

In the studied case, the glass transition temperature for amine-cured materials (*T*_g_ = 50 °C) was 37 °C higher than that of the acid-cured product (*T*_g_ = 13 °C).

When the material is cured with the aromatic amine (at 150 °C), the reaction proceeds to completion without post-curing and yields a fully set, highly stable material with long-lasting properties. This hardener should be selected to create hard, resistant, and stable composites.

Suberic acid as a hardener requires greater precision in the determination of the proper parameters, as the progression of curing is sensitive to the selection of process conditions, significantly affecting the final product properties. In our study, post-curing at 250 °C was required for this hardener to achieve near-complete conversion (after the regular curing route at the initially chosen temperature of 150 °C). The flexibility and sensitivity of epoxy resins cured with this hardener create a prospect for making thermally activated, modern self-healing materials, which suggests promising directions for our future research.

## Figures and Tables

**Table 1:** The curing conditions for both mixtures were measured, with measurement uncertainties as determined by the accuracy of the experimental equipment.

Mixture	Curing Conditions		Post-Curing Treatment
	Temperature [°C]	Time [min]	T_p_ [°C] Heating [10 °C/min]
DKUU/DDM	150 (0, 1)	180 (1)	-
DKUU/SA	150 (0, 1)	180 (1)	250 (0, 1)

**Table 2:** The glass transition temperatures for the plain monomer and both cured products, with measurement uncertainty estimations.

Mixture	T_g_ [°C] from DSC	T_g_ [°C] from BDS
DKUU	−19.3 (0.8)	−27 (23)
DKUU/DDM	50.2 (0.5)	48.9 (6.2)
DKUU/SA	12.5 (0.5)	14.2 (8.9)

## Data Availability

The original contributions presented in this study are included in the article/Supplementary Material. Further inquiries can be directed to the corresponding author.

## Supplementary Materials

The following supporting information can be downloaded at: https://www.mdpi.com/article/10.3390/ma19071358/s1, Figure S1: DSC thermograms of DKUU cured with SA. The graphs show repeatability of the results; Figure S2: DSC thermograms of the curing process (heating route) for DKUU/DDM (a) and DKUU/SA (b), with integration of the melting and curing peaks; Figure S3: DSC thermograms of the curing process (heating route) and the cooling route conducted directly after curing for the mixtures of DKUU cured with DDM (a) and SA (b). Diagrams show repeatability of the results for two independent samples; Figure S4: Representative Havriliak-Negami fits at selected temperatures, along with the values of fit parameters: (a,b) DKUU/DDM, (c,d) DKUU/SA; Figure S5: VFT equation fits of the α-relaxation for neat DKUU (a), DKUU cured with DDM (b), and with SA (c).

## Abbreviations

The following abbreviations are used in this manuscript:

BDS	Broadband Dielectric Spectroscopy
DC	Direct Current
DDM	4,4′-diaminodiphenylmethane
DSC	Differential Scanning Calorimetry
HN	Havriliak–Negami (formula)
SA	Suberic acid
VFT	Vogel–Fulcher–Tammann (formula)
