# Supplementary material for: Tuning the Elastic Properties of Polymer Networks Based on a Selected Biphenyl Epoxy Precursor by Altering the Hardener—Thermal and Dielectric Approach
Source: Materials (Basel). 2026 Mar 29;19(7):1358. doi: 10.3390/ma19071358 (PMC13074072; doi:10.3390/ma19071358)
Supplement: Supplementary file 1 [file materials-19-01358-s001.zip › materials-4051361-supplementary.pdf]

Supplementary Materials

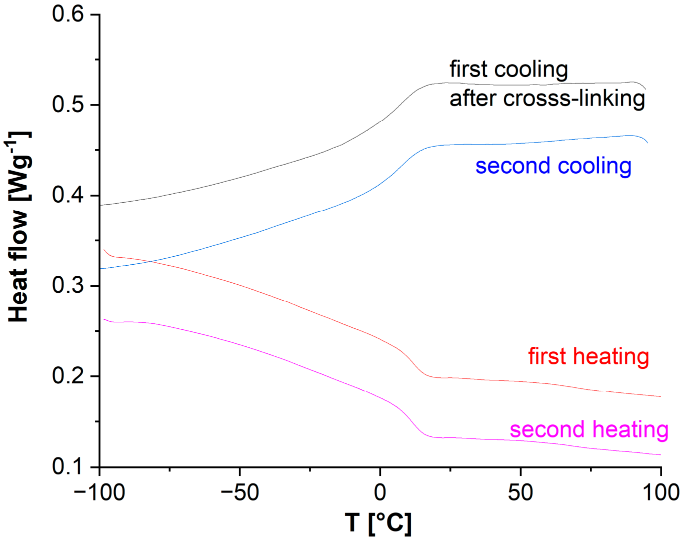

Figure S1. DSC thermograms of DKUU cured with SA. The graphs show repeatability of the results.

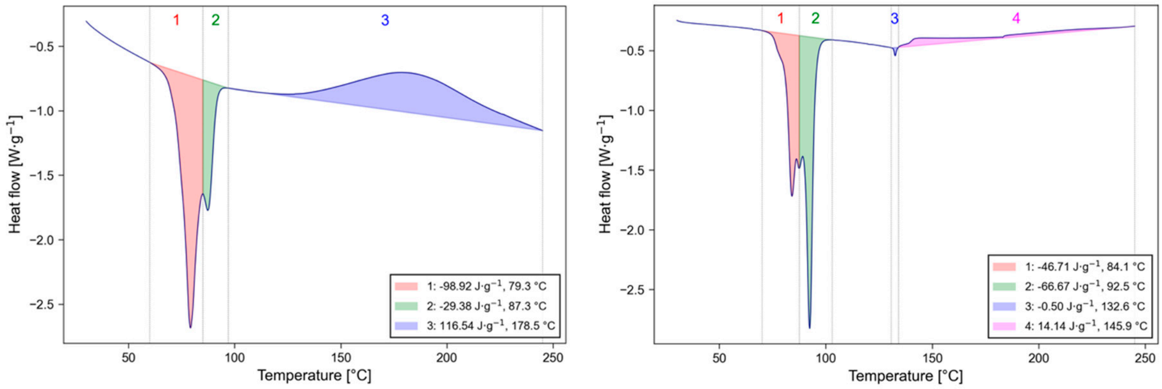

Figure S2. DSC thermograms of the curing process (heating route) for DKUU/DDM (a) and DKUU/SA (b), with integration of the melting and curing peaks.

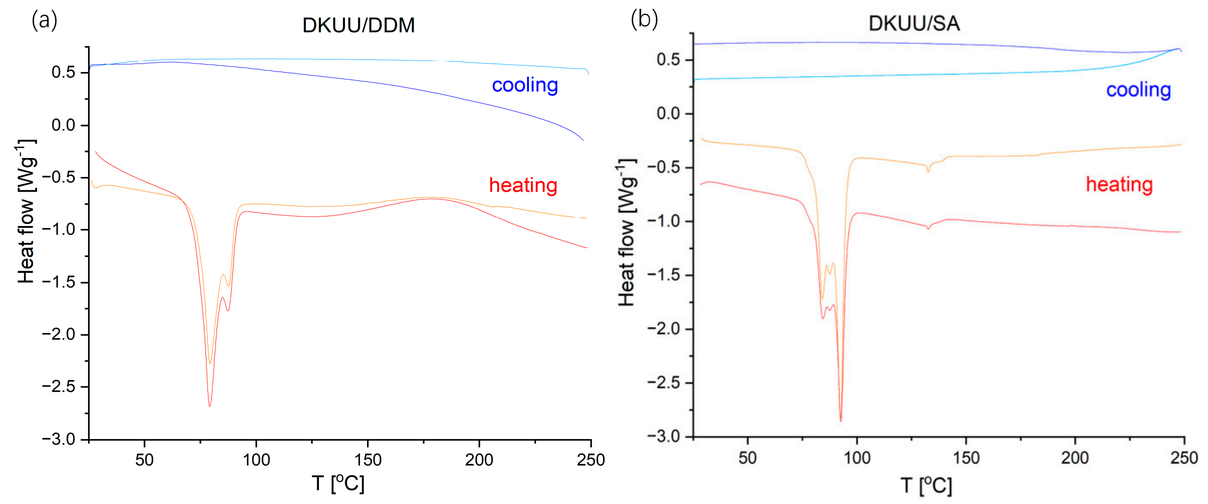

Figure S3. DSC thermograms of the curing process (heating route) and the cooling route conducted directly after curing for the mixtures of DKUU cured with DDM (a) and SA (b). Diagrams show repeatability of the results for two independent samples.

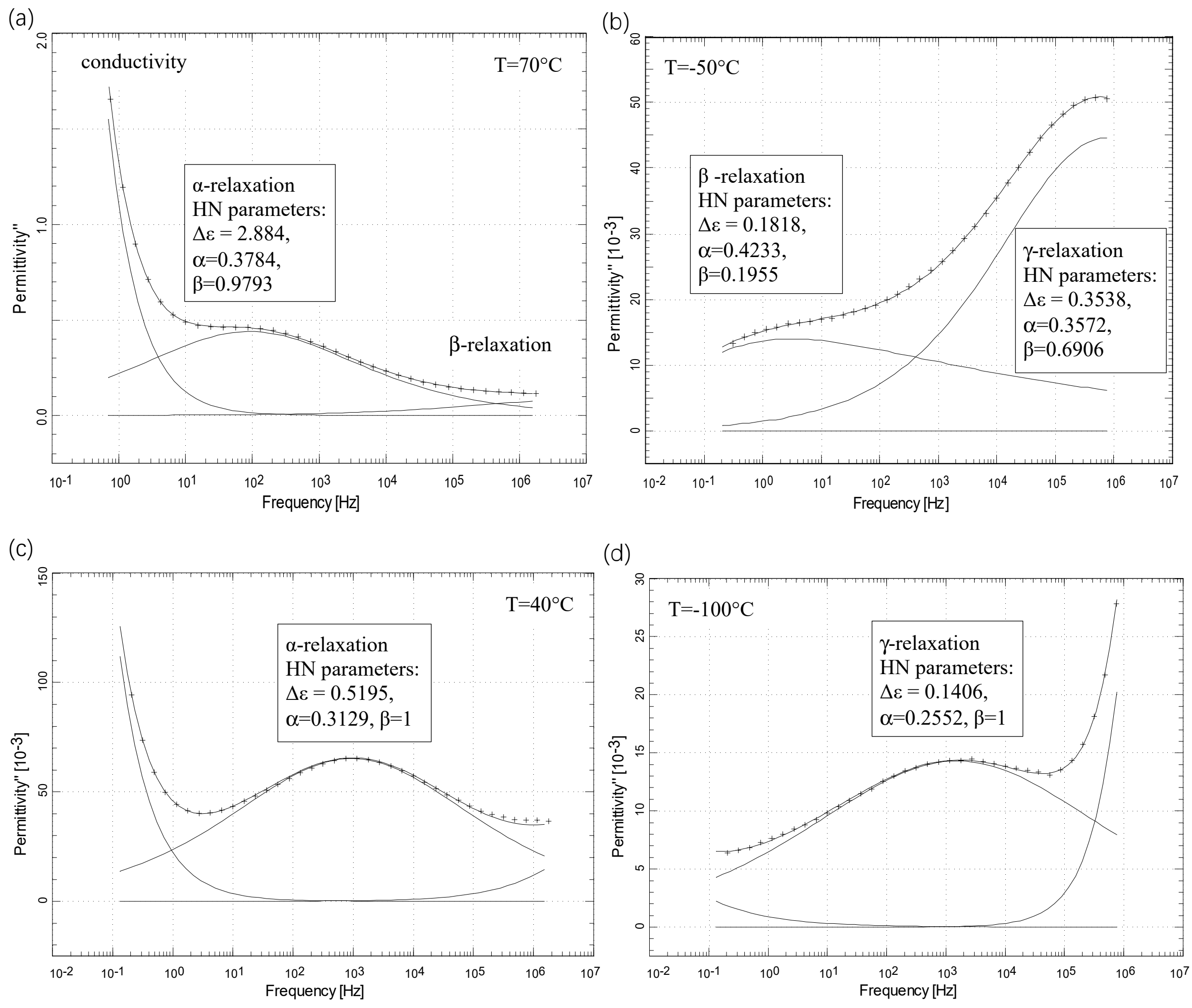

Figure S4. Representative Havriliak-Negami fits at selected temperatures, along with the values of fit parameters:

(a,b) DKUU/DDM, (c,d) DKUU/SA.

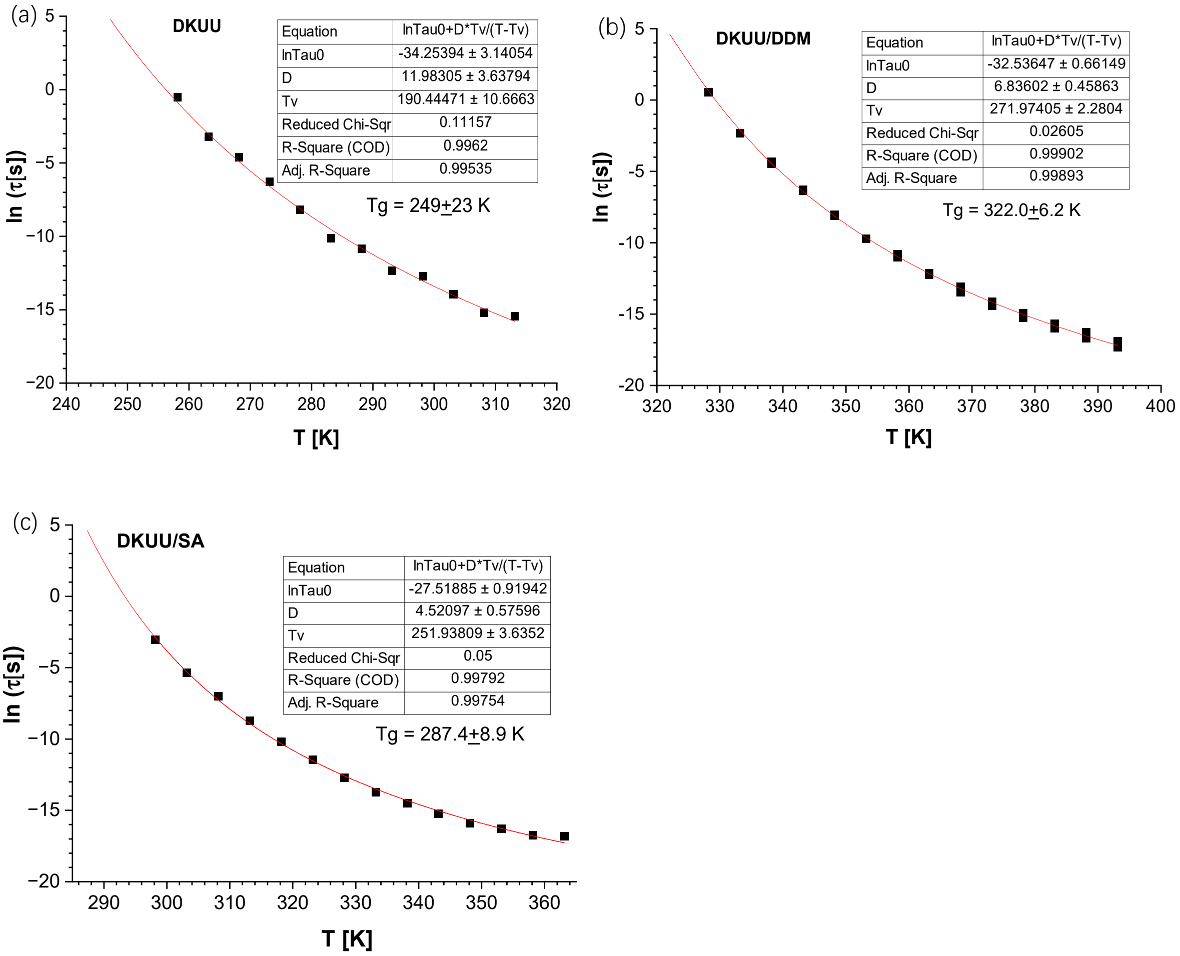

Figure S5. VFT equation fits of the α-relaxation for neat DKUU (a), DKUU cured with DDM (b), and with SA (c).
